# Resource Use and Costs Associated with Coeliac Disease before and after Diagnosis in 3,646 Cases: Results of a UK Primary Care Database Analysis

**DOI:** 10.1371/journal.pone.0041308

**Published:** 2012-07-17

**Authors:** Mara Violato, Alastair Gray, Irini Papanicolas, Melissa Ouellet

**Affiliations:** 1 Department of Public Health, Health Economics Research Centre, University of Oxford, Oxford, United Kingdom; 2 Department of Social Policy, LSE Health, London School of Economics, London, United Kingdom; National Cancer Institute, United States of America

## Abstract

**Background:**

Despite the considerable health impact of coeliac disease (CD), reliable estimates of the impact of diagnosis on health care use and costs are lacking.

**Aims:**

To quantify the volume, type and costs, in a United Kingdom primary care setting, of healthcare resources used by individuals diagnosed with CD up to ten years before and after diagnosis, and to estimate medical costs associated with CD.

**Methods:**

A cohort of 3,646 CD cases and a parallel cohort of 32,973 matched controls, extracted from the General Practice Research Database (GPRD) over the period 1987–2005 were used i) to evaluate the impact of diagnosis on the average resource use and costs of cases; ii) to assess direct healthcare costs due to CD by comparing average resource use and costs incurred by cases vs. controls.

**Results:**

Average annual healthcare costs per patient increased by £310 (95% CI £299, £320) after diagnosis. CD cases experienced higher healthcare costs than controls both before diagnosis (mean difference £91; 95% CI: £86, £97) and after diagnosis (mean difference £354; 95% CI: £347, £361). These differences were driven mainly by higher test and referral costs before diagnosis, and by increased prescription costs after diagnosis.

**Conclusions:**

This study shows significant additional primary care costs associated with coeliac disease. It provides novel evidence that will assist researchers evaluating interventions in this area, and will challenge policymakers, clinicians, researchers and the public to develop strategies that maximise the health benefits of the resources associated with this disease.

## Introduction

Coeliac disease (CD) is an inherited chronic autoimmune disorder that can potentially affect many organ systems beyond the gastrointestinal tract [1], [2], [3]. It is triggered by ingestion of gluten, the protein fraction of wheat, rye and barley. The disease can develop and be diagnosed at any age. The only current available therapy is a lifelong adherence to a gluten-free diet. Prevalence amongst adults and children approaches 1% of the population in international studies but rates of diagnosis are increasing in many countries [4], [5], [6], [7], [8]. Clinical presentation may assume a broad spectrum of symptoms and this often results in delayed or under- diagnosis. Consequently, the disease can be considered a concealed public health problem in various countries [9], [10].

Although the health impact of CD is considerable due to increasing prevalence rates, associated non-specific morbidity and long-term complications [10], little is still known about the resource use and costs to the healthcare system associated with diagnosis of CD. The focus of the existing economic literature has mainly been on estimating the likely costs associated with possible CD screening programmes, or examining differences between diagnostic strategies and technologies, or considering patient-incurred dietary costs [11], [12], [13], [14], [15], [16], [17], [18]. To our knowledge, only two published studies, both conducted in the United States (US), have attempted before and after assessments of the economic impact of CD diagnosis [19], [20]. They both found that an increase in the rate of CD diagnosis was associated with a significant reduction in healthcare services utilisation and costs. However, these US analyses are not generally applicable to the United Kingdom (UK) due to different health systems, patterns of healthcare utilisation and payment mechanisms between countries. Given that no previous cost analysis of CD diagnosis has been carried out in the UK, this study aims to fill the existing gap in the literature by providing new and original evidence on the economic impact of CD in a UK context.

The main objective is to quantify the volume, type and cost of healthcare resources used by individuals diagnosed with CD up to ten years before and after diagnosis, with particular reference to consultations, tests, referrals and prescriptions in the primary care setting where non-acute care is mainly managed, and to compare these with the costs incurred by a matched control-cohort, using the General Practice Research Database (GPRD). Unlike previous published studies and uniquely in the area of coeliac disease research, we also analyse in detail the distribution of prescriptions costs across the 15 British National Formulary (BNF) categories [21] for both the case and control cohorts. We look at data for the period 1987 to 2005.

## Methods

The UK GPRD is one of the largest computerised databases of anonymised longitudinal medical records from primary care. There was, therefore, no need of patients’ consent. GPRD contains data from 1987 onwards and currently covers a population of over 5 million patients throughout the UK. It includes medical diagnostic codes for consultations, tests, referrals and details of prescriptions issued. Data are routinely subject to quality checks in order to guarantee they are ‘up-to-standard’ [22].

### Study Design

We used information supplied by GPRD staff over the period June 1987 to October 2005. Our case-cohort comprised all patients within GPRD with a recorded diagnosis of CD. Following the methods of a previous published study [23], our study population was restricted to those patients with a medical diagnosis of CD according to one of the following Read/OXMIS medical codes (restricted definition of CD): 2690B Coeliac Disease, J690.00 Coeliac Disease, J690z00 Coeliac Disease NOS, J690.13 Gluten Enteropathy, 2690D Infantile Coeliac Disease, J690100 Acquired Coeliac Disease. A control-cohort was identified by selecting ten controls to match each patient with CD by age, gender and general practice. Controls were alive and registered at the practice on the date of the first prospective, up to standard, record of CD for cases. Controls were excluded if they had any record of gluten-free prescription or a non-specific reference to CD, such as gluten-free diet or gluten sensitivity.

A diagnosis date, defined as the date of the first record of CD, was attributed to each patient with CD. Controls were assigned a ‘pseudo-diagnosis’ date coinciding with that of their matched case. To ensure maximum reliability of data we distinguished between ‘incident cases’ – those patients with a date of diagnosis of CD at least 12 months after their up-to-standard record on GPRD began – and ‘prevalent cases’, including all other subjects with CD. Primary analyses made use only of incident cases.

### Data

Details of demographic and diagnostic information were extracted or constructed from the GPRD data for the CD and the control cohorts. They included patient age, gender, smoking status, BMI, Charlson comorbidity score, which, by accounting for both number and severity of comorbidity, classifies patients according to their disease burden [24], [25], [26], length of observation time and the diagnosis/pseudo-diagnosis date by calendar year group.

Information relating to resource use for each patient over the study period was extracted from the GPRD data. It included number of primary care consultations, tests, referrals to out-patient hospital care [27] and prescriptions (by BNF category). Details on the specific composition of each resource use category and the assumptions made for their count [21], [28] can be found in the supplementary material (File S1, Text S1).

For each patient, healthcare costs stratified by subcategory of interest (consultations, tests, referrals and prescriptions) were computed by multiplying units of resource use by their unit costs. These were then summed over all resource use categories to obtain an annual cost and total cost for each patient. Details on the specific unit cost sources [29], [30], [31], [32] and values can be found in the supplementary material (File S1, Table S1). Values were expressed in 2009/10 UK pound sterling (£). When values where available only for 2008/09, prices were adjusted using the Hospital and Community Health Services (HCHS) pay and price inflation index 2009/10 [33].

### Statistical Analysis

We analysed the 10-year period prior to and following diagnosis of CD for cases, and pseudo-diagnosis for the matched control-cohort. We considered the full 20-year period even if data on healthcare utilisation and costs for a case or control subject were available for less than 20 years, provided that data were available on at least some of the matched sets of cases and controls within the study population. Consequently, resource use and costs were calculated on a number of cases/controls that was variable each year over the period of study. Average annual amounts of healthcare resource use and associated costs were calculated and then compared for the identified CD cohort in the 10 years prior to and following diagnosis. The aim was to evaluate the impact of diagnosis on the average resource use and costs incurred by each patient within a primary care setting. Similar analyses were performed for the control-cohort. Analyses were stratified by resource use and cost categories of interest, and by age group at diagnosis (total costs only).

The direct healthcare costs due to CD were estimated by comparing the average annual amounts of healthcare resources use and costs incurred by the CD cohort with the analogous amounts incurred by the matched control-cohort before and after diagnosis/pseudo-diagnosis (for controls) of CD. These analyses were also stratified by resource use and cost category of interest, and by age group at diagnosis/pseudo-diagnosis (total costs only). Similar analyses were conducted for costs of prescriptions by BNF category.

We also investigated whether the widespread introduction of serological testing, approximately between 1993 and 2000 [5], [34], [35], [36], [37], [38], [39] altered the patterns of healthcare utilisation and associated costs in the CD versus non-CD cohorts. A before and after diagnosis comparison of costs for the case and the control cohorts was conducted by stratifying the sample by date of diagnosis/pseudo-diagnosis for each of the cost category of interest.

Consultations, referrals, tests and prescriptions were treated as continuous variables and reported as number of events per patient per year. Resource use rates and mean costs before and after diagnosis were reported together with their standard errors. Rates and mean differences in observed resource use and costs between the two time periods were reported alongside 95% confidence intervals (CI). Statistical differences in mean estimates were evaluated using Student’s two-sided t-test [40]. Unpaired t-tests were used to conform to the decision not to restrict analyses to matched sets for which the ratio ‘cases to controls’ was consistently 1∶10 over the time of the study. All statistical analyses were performed using STATA version 11. Statistical significance was set at *P*-values less than 0.05.

## Results

### Study Population

**Figure 1:**
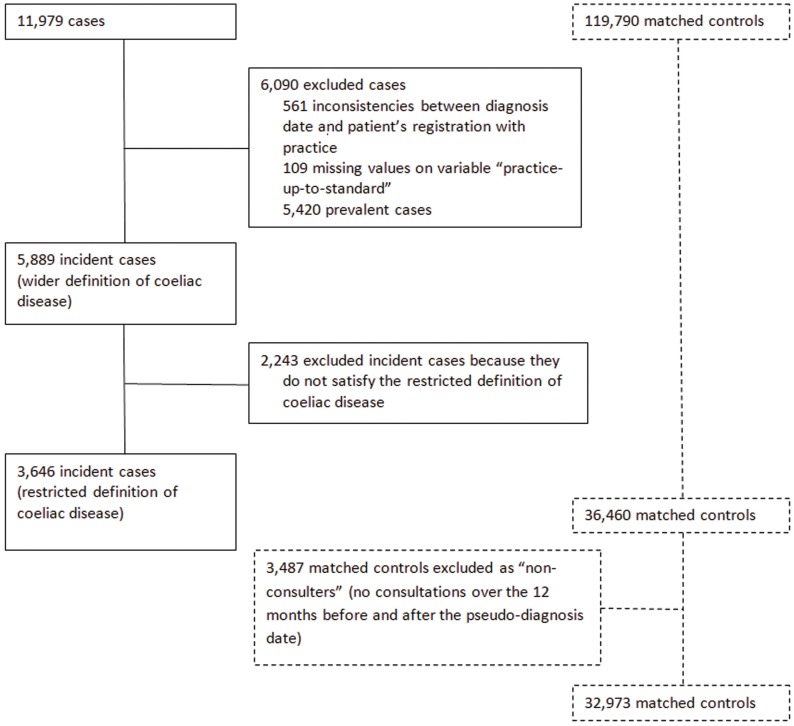
Cases selection profile.

Figure 1 shows the cases selection profile. A cohort of 11,979 cases was initially identified as patients with a medical diagnosis of coeliac disease *or* dermatitis herpetiformis *or* a non-specific reference to coeliac disease *or* a prescription for gluten free products between 1^st^ June 1987 and 31^st^ October 2005 (wider definition of CD). Of these, 561 patients were excluded because of ascertained inconsistencies between the diagnosis date and the patient’s date of registration with the practice. No data on the variable ‘practice-up-to-standard’ were available for further 109 patients, who were excluded. Our decision to focus only on incident cases led to the exclusion of a further 5,420 prevalent cases, reducing the sample to 5,889 patients. A further 2,243 cases were removed because they did not satisfy the more restricted definition of CD. Results are, therefore, based on 3,646 incident cases and the corresponding controls matched on a 1∶10 ratio. A further refinement of the control-cohort was the exclusion of 3,487 individuals as ‘no consulters’ on the grounds that they did not consult over the 12 months before and after the pseudo-diagnosis date.

Table 1 displays demographic and diagnostic information for patients in our study. The 3,646 cases and the 32,973 matched controls contributed 37,208 and 352,205 observed patient years, respectively. The mean observed time was 10.21 years for cases and 10.68 years for controls, with mean pre-diagnosis/pseudo-diagnosis time of 5.01 and 5.41 years for the case and the control cohorts, respectively. The corresponding mean observed time after diagnosis was 5.20 years for cases, and 5.28 years for controls. The mean age at diagnosis of the incident cases was 43.76 years, and 66% of them were women. The percentage of current smokers was higher in the control-cohort than in the CD cohort (19% versus 15%). About 50% of cases were underweight/normal, compared with only 34% among controls. 60% of cases had no comorbid disease, compared with 65% of controls. The difference remained distinct also for low values of the Charlson comorbidity index (25% of cases versus 21% of controls had an index score of 1). Finally, about 61% of cases were diagnosed in 2000 or later. That period followed the widespread adoption of highly sensitive and specific diagnostic serological tests to detect the disease.

**Table 1:** Details on observation time and personal characteristics of coeliac disease cohort and control cohort^1^.

Variables		Coeliac Disease cohort (n = 3,646)	Control cohort (n = 32,973)
**Mean (median) observed time**	**Overall**	10.21 (9.97)^1^	10.68 (10.52)
	**Before diagnosis**	5.01 (4.21)	5.41 (4.67)
	**After diagnosis**	5.20 (4.00)	5.28 (4.12)
**Total observed time (years)** 2		37,208	352,205
**Gender**	**Female**	2,389 (65.52)	22,220 (67.39)
	**Male**	1,257 (34.48)	10,753 (32.61)
**Mean age at beginning of** **observed time (years)**		38.85	38.72
**Age group at beginning of** **observed time (categories)**	**0–3**	367 (10.07)	3,507 (10.64)
	**>3–15**	237 (6.50)	2,080 (6.31)
	**>15–25**	287 (7.87)	2,729 (8.28)
	**>25–35**	644 (17.66)	5,659 (17.16)
	**>35–45**	649 (17.80)	5,715 (17.33)
	**>45–55**	615 (16.87)	5,467 (16.61)
	**>55–65**	452 (12.40)	4,144 (12.57)
	**>65–75**	287 (7.87)	2,709 (8.22)
	**>75**	108 (2.96)	954 (2.89)
**Age at diagnosis or pseudo-** **diagnosis (controls) date**	**Mean**	43.76	44
	**0–18**	576 (15.80)	5,210 (15.80)
	**19–45**	1,248 (34.23)	11,054 (33.52)
	**46 or more**	1,822 (49.97)	16,709 (50.67)
**Smoking status** 3	**Non-smoker**	1,937 (53.12)	15,459 (46.88)
	**Former smoker**	537 (14.73)	4,838 (14.67)
	**Smoker**	552 (15.14)	6,297 (19.10)
	**Unknown**	620 (17.00)	6,379 (19.35)
**BMI** 3	**Underweight/normal**	1,835 (50.33)	11,302 (34.28)
	**Overweight/obese**	874 (23.97)	12,111 (36.73)
	**Unknown**	937 (25.70)	9,560 (28.99)
**Charlson comorbidity score**	**0**	2,188 (60.01)	21,516 (65.25)
	**1**	919 (25.21)	7,105 (21.55)
	**2**	287 (7.87)	2,238 (6.79)
	**3 or more**	249 (6.93)	2,080 (6.31)
	**Unknown**	3 (0.08)	34 (0.10)
**Diagnosis/pseudo-diagnosis year**	**1987–1990**	19 (0.52)	165 (0.50)
	**1991–1993**	308 (8.45)	2,719 (8.25)
	**1994–1996**	407 (11.16)	3,535 (10.72)
	**1997–1999**	695 (19.06)	6,248 (18.95)
	**2000–2003**	1,456 (39.93)	13,235 (40.14)
	**2004–2005**	761 (20.87)	7,071 (21.44)
**Diagnosis/pseudo-diagnosis** **up to 1999 and after**	**1987–1999**	1,429 (39.19)	12,667 (38.42)
	**2000–2005**	2,217 (60.81)	20,306 (61.58)

1 Values are numbers and percentages are presented in parentheses.

2 Matching was performed between cases and controls not on individual years.

3 most recent.

### Impact of Diagnosis of Coeliac Disease on Healthcare Resource Use and Costs

The coeliac cohort had a lower rate of consultations (5.8 vs 6.9), tests (1.9 vs 3) and prescriptions (14.3 vs 40.7) in the 10 year prior to diagnosis than following diagnosis (Table 2). Rate differences were statistically significant. Only the referral rate showed a small but statistically significant decrease between the two periods (0.3 vs 0.21). The rates of resource use for the control-cohort (Table 2) were lower than for case-cohort but, with the exception of referrals, they slightly increased after pseudo-diagnosis. Although statistically significant, the magnitude of these rate differences was almost negligible and more likely driven by secular time trends in healthcare utilisation.

**Table 2:** Resource use and costs: average annual amounts of healthcare resource use and costs per patient in CD versus non-CD cohorts (for a maximum of 10 years before and after diagnosis).

	Cases			Controls			Case-Control Difference		Cases as a proportion of controls	
	Before^1^ (B)	After^1^ (A)	A-B^2^	Before^1^ (B)	After^1^ (A)	A –B^2^	Before^2^	After^2^	Before^2^	After^2^
***RESOURCE USE***										
**Consultations**	5.8 (0.048)	6.9 (0.060)	1.1 (1.006, 1.3)	4.3 (0.012)	4.9 (0.015)	0.65 (0.61, 0.69)	1.5 (1.4, 1.6)	2.01 (1.9, 2.1)	1.35 (1.32, 1.37)	1.41 (1.39, 1.44)
**Tests**	1.9 (0.031)	3 (0.047)	1.1 (0.9, 1.2)	1.1 (0.007)	2.01 (0.011)	0.9 (0.88, 0.93)	0.8 (0.78, 0.88)	0.98 (0.90, 1.05)	1.73 (1.70, 1.81)	1.49 (1.44, 1.54)
**Outpatient referrals**	0.3 (0.005)	0.21 (0.005)	−0.9 (−0.11, −0.08)	0.21 (0.002)	0.19 (0.002)	−0.02 (−0.023,-0.014)	0.09 (0.089, 0.108)	0.02 (0.013, 0.032)	1.43 (1.38, 1.48)	1.11 (1.05, 1.16)
**Prescriptions**	14.3 (0.179)	40.7 (0.341)	26.4 (25.6, 27.1)	11.2 (0.051)	15 (0.076)	3.8 (3.7, 4.01)	3.1 (2.8, 3.4)	25.6 (25.1, 26.1)	1.25 (1.24, 1.31)	2.71 (2.66, 2.76)
***COSTS***										
**Consultations costs**	£164 (1.363)	£184 (1.570)	£20 (£16.4, £24.5)	£120 (0.346)	£130 (0.398)	£10 (£8.8, £10.9)	£43.6 (£41.3, £45.8)	£54.2 (£51.6, £56.7)	1.37 (1.34, 1.39)	1.42 (1.39, 1.44)
**Tests costs**	£9 (0.136)	£13.1 (0.205)	£4.1 (£3.6, £4.6)	£5.3 (0.032)	£8.8 (0.048)	£3.5 (£3.4, £3.6)	£3.7 (£3.5, £4)	£4.3 (£4.01, £4.6)	1.70 (1.65, 1.76)	1.49 (1.44, 1.54)
**Outpatient referrals costs**	£39.2 (0.677)	£27.1 (0.624)	−£12.1(−£13.9, −£10.26)	£26.1 (0.189)	£23.9 (0.205)	−£2.2 (−£2.75, −£1.65)	£13.1 (£12, £14.31)	£3.2 (£2.08, £4.45)	1.50 (1.45, 1.56)	1.13 (1.08, 1.19)
**Prescriptions costs**	£137 (1.743)	£432 (3.614)	£295 (£287, £302)	£106 (0.513)	£142 (0.753)	£36 (£34.3, £37.8)	£30.7 (£27.4, £34.03)	£289 (£284, £294)	1.29 (1.26, 1.33)	3.04 (2.98, 3.10)
**Total costs**	£340 (2.96)	£650 (4.68)	£310 (£299, £320)	£249 (0.79)	£296 (1.07)	£47 (£45, £50 )	£91 (£86, £97)	£354 (£347, £361)	1.37 (1.34, 1.39)	2.20 (2.16, 2.23)

1 Standard error in parentheses.

2 Confidence interval in parentheses.

Total annual healthcare costs increased by £310 (£340 vs £650, 95% CI: £299, £320) in the case-cohort, following CD diagnosis. This 91% increase was mainly driven by the trebling of prescription costs (£ 137 vs £ 432, 95% CI: £ 287, £302). The modest, but statistically significant increase in consultation costs (£ 20, 95% CI: £16.4, £24.5) and test costs (£ 4.1, 95% CI: £3.6, £4.6) was partially offset by the reduction in referral costs (−£12.1, 95% CI:−£13.9,−£10.26). Stratification of total annual healthcare costs by age group at diagnosis (File S1, Table S2) show that total costs per patient more than doubled after diagnosis for the age group ‘46 or more’, whilst it increased by 81% and 84% for age groups ‘0–18′ and ‘19–45′, respectively.

**Figure 2:**
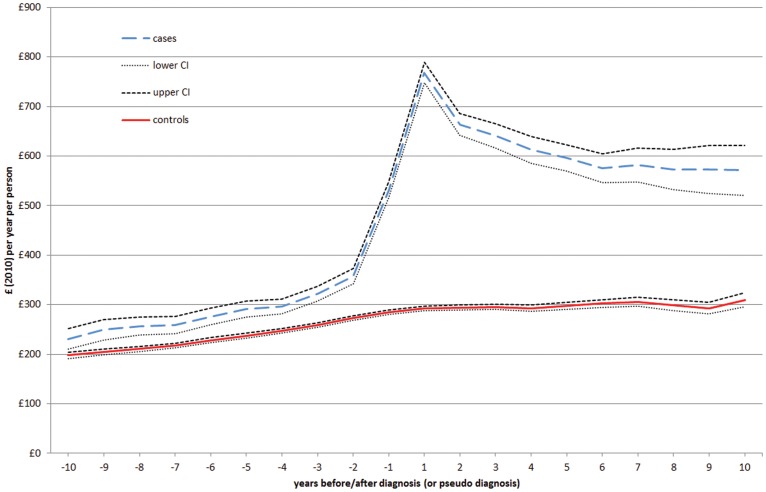
Total costs before and after diagnosis (or pseudo-diagnosis) of coeliac disease.

Figure 2 shows that in the coeliac cohort, the average cost per patient increased gradually until three years before diagnosis (from £231 to £296). Costs continued to increase at a faster rate until a year after diagnosis (up to £768), undergoing a rapid decrease the year after, until they stabilised at around £600 from the sixth year after diagnosis onwards. The average cost per patient for the control-cohort underwent a more modest and gradual increase over the whole 20-year period, from £198 to £310.

**Figure 3:**
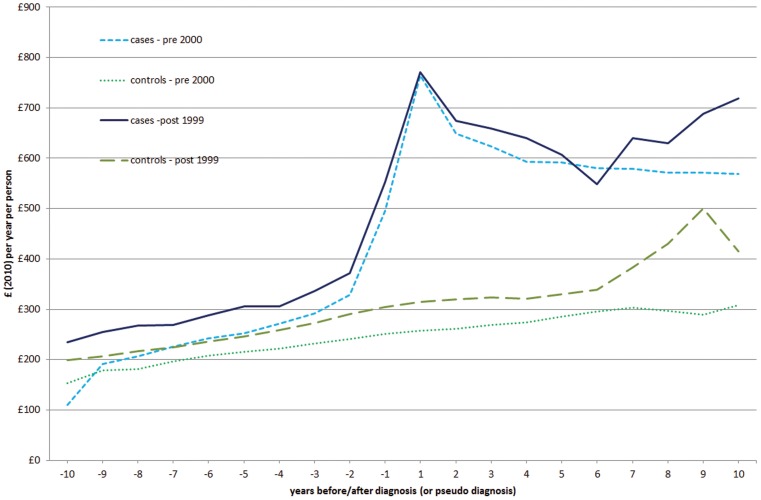
Total costs before and after diagnosis (or pseudo-diagnosis) of coeliac disease. Comparison between PRE-2000 and POST-1999 DIAGNOSIS.

Table 3 shows that, for the majority of BNF categories, the cost of prescriptions for cases was larger than the corresponding costs for controls. The BNF categories more obviously thought to be associated with CD were ‘gastro-intestinal system’ (BNF 1) and ‘food and nutrition’ (BNF 9). Although small in magnitude, the average cost for prescriptions in BNF 1 increased by 38% for the case-cohort (£10.7 vs £ 14.8, 95% CI: £ 3.3, £ 4.8) before and after diagnosis. The control-cohort underwent a corresponding increase of 35%. More strikingly, the average costs for ‘food and nutrition’ (BNF 9) prescriptions (including gluten-free products such as gluten-free bread, pasta, flour, biscuits etc.) for the case-cohort became 25 times larger than in the pre-diagnosis period (£10 vs £246, 95% CI: £ 231, £240) and never returned to the pre-diagnosis levels. The corresponding increase for the controls was 58%. A more detailed analysis focusing exclusively on gluten-free and special diet food supplements revealed that the latter were responsible for 89% of the increased prescriptions costs in BNF 9 after diagnosis (£1.8 vs £220, 95% CI: £214, £222). With respect to the costs for the other BNF categories, our results were consistent with clinical and epidemiological evidence on health problems associated with CD [2], [3], [10]: for instance, the apparently higher prescribing for osteoporosis (BNF 6). The associated prescription costs for the case-cohort increased by 59% for BNF 6, while they increased by 42% for the control-cohort. Growing prescription costs for cases were observed also for BNF categories 3 (respiratory system), 4 (central nervous systems), 11 (eye) [2], [3], [10] and ‘Miscellaneous’ (which included some gluten-free products). Table 4 displays the total and disaggregated healthcare costs for the case and control cohorts stratified by date of diagnosis/pseudo-diagnosis. Figure 3 shows that the pre-2000 cohorts of both cases and controls had lower costs than the corresponding post-1999 cohorts over almost the whole sample period.

**Table 3:** Average annual cost for prescriptions per patient by British National Formulary (BNF) category in CD versus non-CD cohorts (for a maximum of 10 years before and after diagnosis).

	Cases			Controls			Case-Control Difference		Cases as a proportion of controls	
	Before^1^ (B)	After^1^ (A)	A–B^2^	Before^1^ (B)	After^1^ (A)	A–B^2^	Before^2^	After^2^	Before^2^	After^2^
**BNF category**										
**BNF 01: Gastrointestinal**	£10.7 (0.241)	£14.8 (0.303)	£4.1 (£3.3, £4.8)	£5.9 (0.056)	£8 (0.078)	£2.1 (£1.8, £2.2)	£4.8 (£4.4, £5.2)	£6.8 (£6.3, £7.3)	1.81 (1.71, 1.91)	1.85 (1.77, 1.93)
**BNF 02: Cardiovascular**	£11.6 (0.343)	£18 (0.469)	£6.4 (£5.3, £7.5)	£14.1 (0.115)	£23 (0.176)	£8.9 (£8.5, £9.3)	−£2.5 (−£3.2, −£1.7)	−£5 (−£6, −£3.9)	0.82 (0.78, 0.87)	0.78 (0.74, 0.83)
**BNF 03: Respiratory**	£18.1 (0.583)	£26 (0.826)	£7.9 (£5.9, £9.8)	£16.6 (0.182)	£20 (0.218)	£3.4 (£2.8, £3.9)	£1.5 (£0.3, £2.7)	£6 (£4.6, 7.4)	1.09 (1.02, 1.16)	1.30 (1.21, 1.38)
**BNF 04: Nervous system**	£31 (0.678)	£41.4 (1.015)	£10.4 (£8, £12.8)	£25.5 (0.202)	£35.8 (0.303)	£10.3 (£9.6, £11)	£5.5 (£4.2, £6.8)	£5.6 (£3.7, £7.5)	1.22 (1.16, 1.27)	1.16 (1.10, 1.22)
**BNF 05: Infections**	£3.7 (0.054)	£4.1 (0.070)	£0.4 (£0.3, £0.6)	£3 (0.016)	£3.1 (0.019)	£0.1 (£0.04, £0.13)	£0.7 (£0.6, £0.8)	£1 (£0.95, £1.19)	1.23 (1.20, 1.27)	1.32 (1.29, 1.38)
**BNF 06: Endocrinology**	£19.2 (0.444)	£30.5 (0.602)	£11.3 (£9.9, £12.8)	£11.8 (0.108)	£16.7 (0.156)	£4.9 (£4.6, £5.3)	£7.4 (£6.7, £8.1)	£13.8 (£12.8, £14.8)	1.63 (1.54, 1.70)	1.83 (1.75, 1.90)
**BNF 07: Obstetrics & Gynaecology**	£5.4 (0.140)	£5.7 (0.182)	£0.3 (−£0.1, £0.8)	£5.3 (0.046)	£5.5 (0.059)	£0.2 (£0.01, £0.3)	£0.1 (−£0.26, £0.33)	£0.2 (−£0.15, £0.6)	1.02 (0.96, 1.07)	1.04 (0.97, 1.11)
**BNF 08: Malignant disease**	£3.6 (0.327)	£7.5 (0.622)	£3.9 (£2.6, £5.3)	£4 (0.134)	£6.3 (0.186)	£2.3 (£1.8, £2.7)	−£0.4 (−£1.3, £0.42)	£1.2 (£0.09, £2.4)	0.90 (0.73, 1.08)	1.19 (0.99, 1.41)
**BNF 09: Nutrition &** **blood**	£10 (0.253)	£246 (2.276)	£236 (£231, £240)	£2.4 (0.044)	£3.8 (0.071)	£1.4 (£1.2, £1.5)	£7.6 (£7.3, £7.9)	£242 (£240, £243)	4.17 (3.91, 4.42)	64.7 (62.1, 67.3)
**BNF 09: Subset of food supplements** 3	£1.8 (0.083)	£220 (2.16)	£218 (£214, £222)	£0.12 (0.012)	£0.23 (0.021)	£0.11 (£0.07, £0.16)	£1.7 (£1.63, £1.81)	£219 (£218, £221)	15 (12.24, 18.95)	957 (810, 1166)
**BNF 10: Musculoskeletal & joint disease**	£6 (0.143)	£5.8 (0.149)	−£0.2 (−£0.6, £0.19)	£5 (0.041)	£5.84 (0.053)	£0.8 (£0.75, £1)	£1 (£0.7, £1.3)	−£0.04 (−£0.42, £0.24)	1.20 (1.14, 1.25)	0.99 (0.93, 1.04)
**BNF 11: Eye**	£2.3 (0.098)	£4.2 (0.197)	£1.9 (£1.5, £2.3)	£2.2 (0.036)	£3 (0.049)	£0.8 (£0.62, £0.86)	£0.1 (−£0.18, £0.29)	£1.2 (£0.91, £1.54)	1.05 (0.94, 1.13)	1.40 (1.26, 1.53)
**BNF 12: Ear, nose & oropharynx**	£2 (0.059)	£2.7 (0.081)	£0.7 (£0.5, £0.9)	£1.4 (0.016)	£1.7 (0.021)	£0.3 (£0.26, £0.36)	£0.6 (£0.45, £0.66)	£1 (£0.84, £1.11)	1.43 (1.31, 1.48)	1.59 (1.48, 1.68)
**BNF 13: Skin**	£5.8 (0.158)	£7.3 (0.185)	£1.5 (£1, £1.9)	£4.1 (0.036)	£4.7 (0.045)	£0.6 (£0.51, £0.73)	£1.7 (£1.5, £2)	£2.6 (£2.3, £2.9)	1.41 (1.34, 1.50)	1.55 (1.46, 1.63)
**BNF 14: Immunological products**	£1.6 (0.039)	£1.7 (0.050)	£0.1 (−£0.03, £0.2)	£1.46 (0.012)	£1.39 (0.012)	−£0.07(−£0.1,−£0.03)	£0.1 (£0.02, £0.17)	£0.3 (£0.18, £0.34)	1.10 (1.01, 1.12)	1.22 (1.11, 1.26)
**BNF 15: Anaesthesia**	£0.3 (0.041)	£0.5 (0.069)	£0.2 (−£0.01, £0.3)	£0.34 (0.013)	£0.4 (0.018)	£0.06 (£0.03, £0.12)	−£0.04 (−£0.1, £0.06)	£0.1 (−£0.07, £0.17)	0.88 (0.73, 1.22)	1.25 (0.83, 1.54)
**Miscellaneous**	£5.8 (0.239)	£16.1 (0.530)	£10.3 (£9.2, £11.4)	£3.2 (0.060)	£3.3 (0.072)	£0.1 (−£0.05, £0.32)	£2.6 (£2.2, £3)	£12.8 (£12.2, £13.3)	1.81 (1.65, 1.97)	4.88 (4.50, 5.26)
**Total Prescription costs**	£137 (1.743)	£432 (3.614)	£295 (£287, £302)	£106 (0.513)	£142 (0.753)	£36 (£34.3, £37.8)	£30.7 (£27.4, £34.03)	£289 (£284, £294)	1.29 (1.26, 1.33)	3.04 (2.98, 3.10)

1 Standard error in parentheses.

2 confidence interval in parentheses.

3 Gluten-free and special diet products.

**Table 4:** Average annual costs per patient in CD versus non-CD cohorts stratified by date of diagnosis/pseudo-diagnosis date (1987–1999; 2000–2005).

	Cases			Controls			Case-Control Difference		Cases as a proportion of controls	
	Before^1^ (B)	After^1^ (A)	A–B^2^	Before^1^ (B)	After^1^ (A)	A–B^2^	Before^2^	After^2^	Before^2^	After^2^
***Diagnosis/pseudo-diagnosis date: 1987–1999***										
**Consultations costs**	£158 (2.501)	£174 (1.884)	£16 (£9.1, £21.6)	£112 (0.608)	£128 (0.501)	£16 (£14.3, £17.5)	£46 (£42.3, £50.4)	£46 (£42.7, £49.1)	1.41 (1.37, 1.46 )	1.36 (1.33, 1.39)
**Tests costs**	£5.5 (0.182)	£9.4 (0.193)	£3.9 (£3.3, £4.4)	£3.1 (0.039)	£7 (0.054)	£3.9 (£3.7, £4.1)	£2.4 (£2.1, £2.7)	£2.4 (£2, £2.7)	1.77 (1.66, 1.90)	1.34 (1.28, 1.40)
**Outpatient referrals** **costs**	£44.1 (1.247)	£24.6 (0.746)	−£19.5 (−£22.2,−£16.8)	£28.6 (0.361)	£23.8 (0.260)	−£4.8 (−£5.7,−£3.9)	£15.5 (£13.3, £17.7)	£0.8 (−£0.64, £2.33)	1.54 (1.45, 1.64)	1.03 (0.97, 1.10)
**Prescriptions costs**	£126 (2.896)	£419 (4.457)	£293 (£280, £306)	£93.6 (0.846)	£129 (0.887)	£35.4 (£33.2, £38.5)	£32.4 (£27.3, £38.3)	£290 (£284, £296)	1.35 (1.29, 1.42)	3.25 (3.17, 3.33)
**Total costs**	£327 (5.14)	£622 (5.64)	£295 (£278, £312)	£228 (1.34)	£280 (5.14)	£52 (£48, £56)	£99 (£90.6, £108)	£342 (£334, £351)	1.43 (1.39, 1.48)	2.22 (2.14, 2.31)
***Diagnosis/pseudo-diagnosis date: 2000–2005***										
**Consultations costs**	£166 (1.624)	£201 (2.715)	£35 (£28.6, £40.3)	£124 (0.419)	£134 (0.653)	£10 (£8.7, £11.6)	£42 (£39.7, £45.2)	£67 (£62.5, £71)	1.34 (1.31, 1.37)	1.5 (1.45, 1.54)
**Tests costs**	£10.4 (0.174)	£18.9 (0.415)	£8.5 (£7.7, £9.2)	£6.2 (0.042)	£11.5 (0.086)	£5.3 (£5.2, £5.5)	£4.2 (£4, £4.5)	£7.4 (£6.8, £7.9)	1.68 (1.62, 1.74)	1.64 (1.56, 1.71)
**Outpatient referrals** **costs**	£37 (0.805)	£31.4 (1.112)	−£5.6 (−£8.33,−£2.85)	£25 (0.221)	£23.9 (0.335)	−£1.1 (−£1.8,−£0.23)	£12 (£10.7, £13.4)	£7.5 (£5.5, £9.5)	1.48 (1.41, 1.55)	1.31 (1.22, 1.41)
**Prescriptions costs**	£141 (2.143)	£450 (6.07)	£309 (£299, £319)	£111 (0.633)	£162 (1.333)	£51 (£48.1, £53.2)	£30 (£25.7, £33.9)	£288 (£279, £297)	1.27 (1.23, 1.31)	2.78 (2.69, 2.86)
**Total costs**	£345 (3.59)	£692 (8.06)	£347 (£331, £361)	£257 (0.97)	£321 (1.84)	£64 (£60.5, £68)	£88 (£81.8, £94.6)	£371 (£358, £382)	1.34 (1.31, 1.37)	2.16 (2.10, 2.21)

1 Standard error in parentheses.

2 Confidence interval in parentheses.

### Cost Associated with Coeliac Disease

Over the 10-year period preceding diagnosis, the CD cohort experienced significantly higher total costs of £91 compared with the control-cohort (mean total costs: £340 vs £249, 95% CI: £86, £97) (Table 2). This difference was mainly driven by higher costs for tests and referrals (70% and 50%, respectively, compared with matched controls), while consultations and prescriptions costs contributed to a lesser extent (less than 40% higher than for controls). After diagnosis, the difference expanded to £354 (£650 vs £296, 95% CI: £347, £361). This was the results of a trebling of prescriptions costs for cases, an increase in consultations costs (42% higher than for controls) and a decrease in tests and referrals costs (still, however, 49% and 13%, respectively, higher than for controls). All differences reached statistical significance. Analyses stratified by age group (File S1, Table S2) show that over the 10-year period preceding diagnosis the case-control difference in annual total cost per patient was homogeneous across age groups ‘19–45’ and ‘46 or more’, but 13% higher for the age group ‘0-18’ (£241 vs £139, 95% CI: £93, £111). After diagnosis, the costs difference between cases and controls were similar for age groups ‘0–18’ and ‘19–45’, but resulted 29% higher for age group ‘46 or more’ (£810 vs £409, 95% CI: £390, £413).

A closer look at the prescription costs (Table 3) reveals that the case-cohort made a significantly higher use of drugs in BNF categories 1 (gastrointestinal), 6 (endocrinology) and 9 (nutrition and blood) both before and after diagnosis. The pre-diagnosis prescription costs for the cohort of cases for BNF 1 and 6 were almost double compared with the costs incurred by the control-cohort; costs for BNF 9 were quadruple, and when focusing only on the subset of prescriptions for food supplements (gluten-free and special diet products) costs became 15 times higher. After diagnosis, the proportion of costs of cases to controls remained almost unchanged for BNF 1 and 6 but was 65 times higher for cases than for controls for BNF category 9. For the other BNF categories for which cost differences between case and control cohorts were statistically significant, the higher costs for cases compared with controls ranged from 9% (BNF 3; respiratory) to 41% (BNF 13: skin) before diagnosis, and from 16% (BNF 4: nervous system) to 59% (BNF 12: ear, nose & oropharynx), after diagnosis.

Table 4 stratifies healthcare costs by diagnosis date. Comparisons of the stratified analyses with those run on the whole sample period (Table 2) indicate that cost differences between cases and controls were larger before diagnosis and smaller after diagnosis in the period up to 1999, whilst in the post-1999 period that situation was reversed.

## Discussion

To the best of our knowledge, this is the first detailed evaluation of the resource use and costs before and after diagnosis of CD in the primary care setting of the UK healthcare system.

Average per-patient annual healthcare costs in primary care significantly increased by 91% for CD patients after they had been diagnosed with the disease. The trend of increased expenditure was stronger in the immediate pre- and post-diagnosis period, which is consistent with clinical practice (Figure 2). Diagnosis usually follows a period of more intense utilisation of healthcare, of which it is the outcome, and it is also followed by an equivalent, if not larger, use of healthcare resources [8] after diagnosis, as patients may undergo screening for comorbid conditions, such as vitamin and mineral deficiencies, anaemia and osteoporosis. Such activity, however, reduces sharply during the subsequent years and in our data stabilised about six years after diagnosis, but at a level much higher than the pre-diagnosis average cost. Analyses of average per-patient annual healthcare costs stratified by age group at diagnosis also showed that diagnosis in older individuals (46 or more) resulted in higher costs than diagnosis in young (0–18) and younger adults (19–45). Diagnosis earlier in the life of an individual, therefore, may help reduce healthcare costs.

This first result seems, at first glance, in clear contrast with the sparse previously published empirical evidence. Previous studies [19], [20] found that direct medical total costs reduced significantly for CD patients after diagnosis. Those investigations, however, were carried out in the US, which has a very different healthcare system from the UK NHS, and in particular where costs of pharmaceutical prescriptions were not covered. In the UK, prescription medicines are mainly prescribed by NHS doctors and are typically taken to a pharmacy to be dispensed. In England, a prescription fee of £7.40 per item is charged (as of 1 April 2011) [41], although the elderly, those on low incomes and patients with chronic diseases are exempt; prescription charges have been completely abolished in Wales [42], Scotland [43] and Northern Ireland [44]. Consequently, the US finding of a reduction in medical costs following CD diagnosis and treatment may hide a shift of the economic costs to patients, as the additional costs of adhering to a gluten-free diet are not reimbursed to the patients, in contrast to the UK, where individuals diagnosed with CD are prescribed gluten-free products. The results of our study corroborate this hypothesis.

Similarly to the US results, coeliac patients in our study underwent a post-diagnosis reduction in referral costs. This partially counterbalanced the slightly higher level of costs for consultations and tests (but not prescriptions) in the post-diagnosis period. Increased consultations and tests may occur because CD patients have to be checked at regular intervals by a healthcare team, including a GP and a dietician, in order to monitor adherence to a gluten-free diet and to reinforce the importance of such compliance [38]. Additionally, higher rates of consultations and tests may be due to comorbidities associated with CD.

Differently from the US studies, our analyses included costs of prescriptions, which were found to be the major driver of the increase in post-diagnosis CD costs. Noticeably, prescriptions costs for BNF category 9 ‘Nutrition and blood’, which includes gluten-free products, underwent a 25-fold increase from an average annual pre-diagnosis cost of £10 to an average of £246 post-diagnosis per coeliac patient, 89% of the latter cost represented by prescriptions for food supplements (gluten-free and special diet products).

Increased post-diagnosis prescription costs for CD patients were also identified in other BNF categories (e.g. gastrointestinal, respiratory, nervous systems, endocrinology etc). This may represent further evidence on the cumulative morbidity experienced by CD patients compared with the general population [39]. A variety of health problems mainly due to untreated disease, and which can persist in the absence of compliance with a gluten-free diet, has been discussed in previous studies [10], [39].

The stratification of healthcare costs by calendar year of diagnosis indicated that, on average, cases cost 43% more than controls before diagnosis up to 1999, compared with 34% more in the post-1999 period. After diagnosis, the difference remained almost unchanged. This is compatible with the hypothesis that improved methods for detecting CD – notably wide adoption of serological testing – and a higher awareness of the disease and its symptoms among health professionals [2], led to a reduction in the number of consultations and tests performed prior to diagnosis.

In evaluating the healthcare costs attributable to CD prior to diagnosis, we found that the average cost per patient with undetected and untreated CD was 37% higher than the corresponding cost for a patient in the control-cohort. Cost comparisons between cases and controls up to 10 years after diagnosis showed that diagnosis and treatment of CD did not reduce direct healthcare cost in aggregate, but did reduce costs of tests (by 21%) and referrals (by 37%).

Our estimates of the costs of undiagnosed and diagnosed CD will help inform clinical and public health policy, facilitate the economic evaluation of diagnostic and treatment interventions, complement the existing NICE guidelines on the management of CD [7], and may increase policymakers’ awareness of the potential costs savings deriving from early identification of CD.

Other strengths of this study include the large sample size, the time frame of 20 years, which was considerably longer than those adopted in previous published studies [19], [20], the wide geographical coverage of the study population, the accuracy and reliability of the GPRD dataset in a primary care setting and the richness of clinical details included in it. These features allowed us to obtain an accurate classification of the patients included in our study, and to produce results that we believe are broadly generalisable to the whole population of the UK. The use of the GPRD posed many challenges to our analyses, notably difficulties in processing such a large and extremely complex dataset, and making full use of all the relevant clinical and pharmaceutical products codes. Only a small number of studies have previously used the GPRD for studying CD, mainly from an epidemiological perspective [23], [45]. Our use of the database to evaluate the economic implications of CD diagnosis emerges, therefore, as novel.

Several limitations of our study should be noted. Firstly, we used a fixed unit cost for each resource category during the whole 20-year period of our analyses. Although this is a standard practice in cost studies and was dictated by the lack of a long time series of unit costs, it might result in our total costs being slightly overestimated. Secondly, although cases and controls were identified on a ratio of 1∶10 and matched by age, sex and practice, we did not match by registration year. We also discarded single individuals if they had missing values in some of the relevant variables. Analyses were therefore conducted on a variable number of cases and controls over the duration of the study and did not strictly follow a matched case-control design. However, we consider it unlikely that this will have had a significant impact on our results, given the sample sizes involved. Thirdly, whilst GPRD is an accurate source of resource use data in the primary care context, the capture of data for secondary care resource use is, by the intrinsic nature of the dataset, less precise, especially with respect to inpatient episodes and follow-up outpatient visits. We have consequently not attempted to include these, and only include initial outpatient referrals from the GP to secondary care. GPRD now permits linkage of patient records to the Hospital Episodes Statistics database, and this will permit future research to more fully capture the primary-secondary care interface and reliably quantify hospital use in the coeliac and non-coeliac population. Fourthly, we focused only on the evaluation of direct costs incurred or initiated by NHS primary care services and did not include indirect costs associated with reduced productivity caused by impaired ability to work and absence from work, out-of-pocket costs such as those incurred in following a special and expensive diet, and other costs to those with coeliac disease and their families.

In addition to economic costs, CD also imposes a significant impact on quality of life [34], [46], [47], [48], [49], [50], [51], [52], [53]. Some studies have shown that the symptoms of undiagnosed CD are associated with a prolonged and substantial decrement of quality of life [34], while long-term dietary compliance generally improves quality of life [50], [52]. However, other studies have suggested that, even after years of compliance with a gluten-free diet, many patients with CD consider it a significant inconvenience [46], [47], [48], [49], [51], [53]. Our results need to be interpreted in light of these findings: new strategies to diagnose and treat CD will impact not only on direct healthcare costs, but also the quality of life of patients.

In conclusion, this study provides a uniquely detailed description of healthcare costs before and after diagnosis of CD in the UK healthcare system. By identifying and quantifying the relative contribution of different types of resource use to the primary care costs of each CD patient, and comparing these to carefully matched controls, this study may provide insights into the potential cost impact and cost-effectiveness of new ways of detecting and treating CD.

## Supporting Information

Table S1 **Unit costs.**
(PDF)

Table S2 **Total costs per patient by age group in CD versus non-CD cohorts (for a maximum of 10 years before and after diagnosis).**
(PDF)

Text S1 **Specific composition of each resource use category and assumptions made for their count.**
(PDF)

## References

[1] Fernandez A, Gonzalez L, de-la-Fuente J (2010). Coeliac disease: clinical features in adult populations.. Revista espanola de enfermedades digestivas : organo oficial de la Sociedad Espanola de Patologia Digestiva.

[2] Green PH, Cellier C (2007). Celiac disease.. The New England journal of medicine.

[3] van Heel DA, West J (2006). Recent advances in coeliac disease.. Gut.

[4] Bingley PJ, Williams AJ, Norcross AJ, Unsworth DJ, Lock RJ (2004). Undiagnosed coeliac disease at age seven: population based prospective birth cohort study.. BMJ.

[5] Green PHR, Jabri B (2003). Coeliac disease.. Lancet.

[6] Mustalahti K, Catassi C, Reunanen A, Fabiani E, Heier M (2010). The prevalence of celiac disease in Europe: results of a centralized, international mass screening project.. Annals of medicine.

[7] NICE (2009). Coeliac disease.. Recognition and assessment of coeliac disease.

[8] West J, Logan RF, Hill PG, Lloyd A, Lewis S (2003). Seroprevalence, correlates, and characteristics of undetected coeliac disease in England.. Gut.

[9] Byass P, Kahn K, Ivarsson A (2011). The global burden of childhood coeliac disease: a neglected component of diarrhoeal mortality?. Plos One.

[10] Mearin ML, Ivarsson A, Dickey W (2005). Coeliac disease: is it time for mass screening?. Best practice & research Clinical gastroenterology.

[11] Dorn SD, Matchar DB (2008). Cost-effectiveness analysis of strategies for diagnosing celiac disease.. Digestive Diseases and Sciences.

[12] Harewood GC (2004). Economic comparison of current endoscopic practices: Barrett’s surveillance vs. ulcerative colitis surveillance vs. biopsy for sprue vs. biopsy for microscopic colitis.. Digestive Diseases and Sciences.

[13] Harewood GC, Murray JA (2001). Diagnostic approach to a patient with suspected celiac disease: a cost analysis.. Digestive Diseases and Sciences.

[14] Lee AR, Ng DL, Zivin J, Green PH (2007). Economic burden of a gluten-free diet.. Journal of human nutrition and dietetics : the official journal of the British Dietetic Association.

[15] Mein SM, Ladabaum U (2004). Serological testing for coeliac disease in patients with symptoms of irritable bowel syndrome: a cost-effectiveness analysis.. Alimentary pharmacology & therapeutics.

[16] Spiegel BM, DeRosa VP, Gralnek IM, Wang V, Dulai GS (2004). Testing for celiac sprue in irritable bowel syndrome with predominant diarrhea: a cost-effectiveness analysis.. Gastroenterology.

[17] Stevens L, Rashid M (2008). Gluten-free and regular foods: a cost comparison.. Canadian journal of dietetic practice and research : a publication of Dietitians of Canada  =  Revue canadienne de la pratique et de la recherche en dietetique : une publication des Dietetistes du Canada.

[18] Yagil Y, Goldenberg I, Arnon R, Ezra V, Ashkenazi I (2005). Serologic testing for celiac disease in young adults-a cost-effect analysis.. Digestive Diseases and Sciences.

[19] Green PH, Neugut AI, Naiyer AJ, Edwards ZC, Gabinelle S (2008). Economic benefits of increased diagnosis of celiac disease in a national managed care population in the United States.. Journal of insurance medicine.

[20] Long KH, Rubio-Tapia A, Wagie AE, Melton LJ, 3rd, Lahr BD, et al (2010). The economics of coeliac disease: a population-based study.. Alimentary pharmacology & therapeutics.

[21] British National Formulary BNF Categories..

[22] Hollowell J (1997). The General Practice Research Database: quality of morbidity data.. Population trends.

[23] West J, Logan RF, Smith CJ, Hubbard RB, Card TR (2004). Malignancy and mortality in people with coeliac disease: population based cohort study.. BMJ.

[24] Charlson ME, Pompei P, Ales KL, MacKenzie CR (1987). A new method of classifying prognostic comorbidity in longitudinal studies: development and validation.. Journal of chronic diseases.

[25] Deyo RA, Cherkin DC, Ciol MA (1992). Adapting a clinical comorbidity index for use with ICD-9-CM administrative databases.. Journal of clinical epidemiology.

[26] Khan NF, Perera R, Harper S, Rose PW (2010). Adaptation and validation of the Charlson Index for Read/OXMIS coded databases.. BMC family practice.

[27] Hodgson C, Ellis C (2001). Time trends in GP outpatient referrals.. Health Statistics Quartely 10, Office for National Statistics.

[28] Rowlands S, Moser K (2002). Consultation rates from the general practice research database.. The British journal of general practice : the journal of the Royal College of General Practitioners.

[29] Department of Health (2010). National Schedule of Reference Costs Year : ‘2009–10’..

[30] (2009). The information centre NHS Prescription cost analysis, England-2.

[31] NICE (2009). Etanercept, Infliximab and Adalimumab for the Treatment of Psoriatic Arthritis: a Systematic Review and Economic Evaluation..

[32] Personal Social Services Research Unit (2010). Unit Costs of Health and Social care 2009..

[33] Department of Health (2010). Hospital and Community Health Services (HCHS) pay and price inflation index 2009/10..

[34] Gray AM, Papanicolas IN (2010). Impact of symptoms on quality of life before and after diagnosis of coeliac disease: results from a UK population survey.. Bmc Health Services Research 10.

[35] Corrao G, Corazza GR, Bagnardi V, Brusco G, Ciacci C (2001). Mortality in patients with coeliac disease and their relatives: a cohort study.. Lancet.

[36] Logan RF, Rifkind EA, Turner ID, Ferguson A (1989). Mortality in celiac disease.. Gastroenterology.

[37] Nielsen OH, Jacobsen O, Pedersen ER, Rasmussen SN, Petri M (1985). Non-tropical sprue. Malignant diseases and mortality rate.. Scandinavian Journal of Gastroenterology.

[38] Rostom A, Murray JA, Kagnoff MF (2006). American Gastroenterological Association (AGA) Institute technical review on the diagnosis and management of celiac disease.. Gastroenterology.

[39] Rubio-Tapia A, Kyle RA, Kaplan EL, Johnson DR, Page W (2009). Increased prevalence and mortality in undiagnosed celiac disease.. Gastroenterology.

[40] Armitage P, Berry G, Matthews J (2002). Statistical Methods in Medical Research.. Oxford: Blackwell Publishing.

[41] NHS England (2011). Prescription costs..

[42] Welsh Government (2006). Free prescriptions in Wales..

[43] Scottish Government (2011). NHS Prescriptions..

[44] NHS (2008). Northern Ireland Prescriptions..

[45] West J, Logan RF, Card TR, Smith C, Hubbard R (2003). Fracture risk in people with celiac disease: a population-based cohort study.. Gastroenterology.

[46] Ciacci C, D’Agate C, De Rosa A, Franzese C, Errichiello S (2003). Self-rated quality of life in celiac disease.. Digestive Diseases and Sciences.

[47] Hallert C, Granno C, Grant C, Hulten S, Midhagen G (1998). Quality of life of adult coeliac patients treated for 10 years.. Scandinavian Journal of Gastroenterology.

[48] Hallert C, Granno C, Hulten S, Midhagen G, Strom M (2002). Living with coeliac disease: controlled study of the burden of illness.. Scandinavian Journal of Gastroenterology.

[49] Hauser W, Stallmach A, Caspary WF, Stein J (2007). Predictors of reduced health-related quality of life in adults with coeliac disease.. Alimentary pharmacology & therapeutics.

[50] Hershcovici T, Leshno M, Goldin E, Shamir R, Israeli E (2010). Cost effectiveness of mass screening for coeliac disease is determined by time-delay to diagnosis and quality of life on a gluten-free diet.. Alimentary pharmacology & therapeutics.

[51] Usai P, Manca R, Cuomo R, Lai MA, Boi MF (2007). Effect of gluten-free diet and co-morbidity of irritable bowel syndrome-type symptoms on health-related quality of life in adult coeliac patients.. Digestive and liver disease : official journal of the Italian Society of Gastroenterology and the Italian Association for the Study of the Liver.

[52] Viljamaa M, Collin P, Huhtala H, Sievanen H, Maki M (2005). Is coeliac disease screening in risk groups justified? A fourteen-year follow-up with special focus on compliance and quality of life.. Alimentary pharmacology & therapeutics.

[53] Whitaker JK, West J, Holmes GK, Logan RF (2009). Patient perceptions of the burden of coeliac disease and its treatment in the UK.. Alimentary pharmacology & therapeutics.

