# Supplementary material for: Resource Use and Costs Associated with Coeliac Disease before and after Diagnosis in 3,646 Cases: Results of a UK Primary Care Database Analysis
Source: PLoS One. 2012 Jul 17;7(7):e41308. doi: 10.1371/journal.pone.0041308 (PMC3398900; doi:10.1371/journal.pone.0041308)
Supplement: Table S1 — Unit costs. (PDF) [file pone.0041308.s001.pdf]

Supporting Information S1 - Table S1

Title of the article: **Resource use and costs associated with coeliac disease before and after diagnosis in 3,646 cases: results of a UK primary care database analysis**

Table S1 - Unit costs[1]

Item	£s	Source	Notes
Consultations:			
GP consultation in surgery	£31.20	Personal Social Services Research Unit. Unit Costs of Health and Social Care 2009. University of Kent, 2010.	Table 8.8b. Cost including qualifications, excluding other direct care staff costs.
GP consultation by telephone	£ 19.10	Personal Social Services Research Unit. Unit Costs of Health and Social Care 2009. University of Kent, 2010.	Table 8.8b. Cost including qualifications, excluding other direct care staff costs.
Nurse consultation in surgery	£ 11.07	Personal Social Services Research Unit. Unit Costs of Health and Social Care 2009. University of Kent, 2010.	Table 8.6. Cost including qualifications
GP/nurse consultation at home/out-of-hours	£ 105.63 (GP) £20.12 (nurse)	Personal Social Services Research Unit. Unit Costs of Health and Social Care 2009. University of Kent, 2010.	Weighted average of GP consultations (*at £105 per consultation; Table 8.8b) and nurse consultations (* at £20 per consultation; Table 8.6).
Referrals:			
Referral to an out-patient department: Gastroenterology (A)	£ 168.25	National Schedule of Reference Costs Year : '2009-10'. http://www.dh.gov.uk/en/Publicationsandstatistics/Publications/PublicationsPolicyAndGuidance/DH_123459 accessed 11/02/11	National Schedule of Reference Costs Year: '2009-10' - NHS Trusts Consultant Led: First Attendance Non-Admitted Face to Face. (A): Service codes 251, 301M
Referral to an out-patient department: all but Gastroenterology (A), Obstetrics (B), Genito-urinary (C), X-ray (D), Pathology (E), Accident & Emergency (F)	£124.63	As above.	As above. Weighted average of all types but (A), (B), (C), (D), (E), (F). - (A): Service codes 251, 301M - (B): Service code 501 - (C): Service codes 101, 211, 360 - (D): Service code 812 - (E): Service codes 252, 253, 255, 302, 303, 311, 313, 317 - (F): Service code 180

Tests:			
Full blood count	£ 2.76	NICE. Etanercept, Infliximab and Adalimumab for the Treatment of Psoriatic Arthritis: a Systematic Review and Economic Evaluation. NICE 2009. http://www.nice.org.uk/nicemedia/live/11966/47855/47855.pdf accessed 11/02/11	Table 10.13.2 - 2009 prices.
Liver function test	£ 0.69	As above.	Table 10.13.2
Electrolytes	£ 1.28	As above.	Table 10.13.2
Tests- other	£ 4.85	As above.	Table 10.13.2 - Average of the unit costs of all the 'Laboratory tests' excluding Full blood count, Liver function test, Electrolytes and Chest-X ray
Prescriptions:			
Prescriptions by British National Formulary (BNF) category:			
BNF 01: Gastro-intestinal system	£ 6.45	The information centre. NHS. Prescription cost analysis, England-2009. http://www.ic.nhs.uk/statistics-and-data-collections/primary-care/prescriptions/prescription-cost-analysis-england--2009 accessed 11/02/11	TAB: Totals for BNF Chapters - Nic/Pxs (£)
BNF 02: Cardiovascular system	£ 5.91	As above.	TAB: Totals for BNF Chapters - Nic/Pxs (£)
BNF 03: Respiratory system	£ 17.12	As above.	TAB: Totals for BNF Chapters - Nic/Pxs (£)
BNF 04: Central nervous system	£ 11.60	As above.	TAB: Totals for BNF Chapters - Nic/Pxs (£)
BNF 05: Infections	£ 4.24	As above.	TAB: Totals for BNF Chapters - Nic/Pxs (£)
BNF 06: Endocrine system	£ 12.45	As above.	TAB: Totals for BNF Chapters - Nic/Pxs (£)
BNF 07: Obstetrics, gynaecology, & urinary-tract disorders	£ 14.80	As above.	TAB: Totals for BNF Chapters - Nic/Pxs (£)
BNF 08: Malignant disease & immunosuppression	£ 91.01	As above.	TAB: Totals for BNF Chapters - Nic/Pxs (£)

BNF 09: Nutrition & blood	£ 11.42	As above.	TAB: Totals for BNF Chapters - Nic/Pxs (£)
BNF 10: Musculoskeletal & joint diseases	£ 6.28	As above.	TAB: Totals for BNF Chapters - Nic/Pxs (£)
BNF 11: Eye	£ 8.50	As above.	TAB: Totals for BNF Chapters - Nic/Pxs (£)
BNF 12: Ear, nose & oropharynx	£ 6.80	As above.	TAB: Totals for BNF Chapters - Nic/Pxs (£)
BNF 13: Skin	£ 6.77	As above.	TAB: Totals for BNF Chapters - Nic/Pxs (£)
BNF 14: Immunological products & vaccines	£ 9.08	As above.	TAB: Totals for BNF Chapters - Nic/Pxs (£)
BNF 15: Anaesthesia	£ 7.84	As above.	TAB: Totals for BNF Chapters - Nic/Pxs (£)
Miscellaneous	£ 9.70	As above.	TAB: Overall total Nic/Pxs (£)

[1] **All costs in 2009/10 prices. Values available only in 2008/09 prices were adjusted for the Hospital and Community Health Services (HCHS) pay and price inflation 2009/10 (2009/10 value = 0.6 %)**
