# Supplementary material for: Resource Use and Costs Associated with Coeliac Disease before and after Diagnosis in 3,646 Cases: Results of a UK Primary Care Database Analysis
Source: PLoS One. 2012 Jul 17;7(7):e41308. doi: 10.1371/journal.pone.0041308 (PMC3398900; doi:10.1371/journal.pone.0041308)
Supplement: Table S2 — Total costs per patient by age group in CD versus non-CD cohorts (for a maximum of 10 years before and after diagnosis). (PDF) [file pone.0041308.s002.pdf]

Supporting Information S1 - Table S2

Title of the article: Resource use and costs associated with coeliac disease before and after diagnosis in 3,646 cases: results of a UK primary care database analysis

Table S2– Total costs per patient by age group in CD versus non-CD cohorts (for a maximum of 10 years before and after diagnosis)

	Cases			Controls			Case-Control Difference		Cases as a proportion of controls	
	Before[1] (B)	After[1] (A)	A-B[2]	Before[1] (B)	After[1] (A)	A –B[2]	Before[2]	After[2]	Before[2]	After[2]
**Total costs**										
**0-18 years**	£241 (6.58)	£436 (8.00)	£195 (£173, £217)	£139 (1.37)	£122 (1.07)	-£17 (-£20, -£13)	£102 (£93, £111)	£314 (£306, £322)	1.73 (1.64, 1.83)	3.57 (3.43, 3.72)
**19-45 years**	£283 (4.50)	£522 (6.76)	£239 (£222, £254)	£193 (1.10)	£212 (1.40)	£19 (£15, £22)	£90 (£83, £98)	£310 (£301, £319)	1.47 (1.42, 1.52)	2.46 (2.39, 2.53)
**46 or more years**	£387 (4.21)	£810 (7.32)	£423 (£408, £439)	£297 (1.17)	£409 (1.76)	£112 (£108, £116)	£90 (£82, £98)	£401 (£390, £413)	1.30 (1.27, 1.33)	1.98 (1.94, 2.02)
**All ages**	£340 (2.96)	£650 (4.68)	£310 (£299, £320)	£249 (0.79)	£296 (1.07)	£47 (£45, £50)	£91 (£86, £97)	£354 (£347, £361)	1.37 (1.34, 1.39)	2.20 (2.16, 2.23)

[1]Standard error in parentheses

[2]Confidence interval in parentheses
