# Supplementary material for: Resource Use and Costs Associated with Coeliac Disease before and after Diagnosis in 3,646 Cases: Results of a UK Primary Care Database Analysis
Source: PLoS One. 2012 Jul 17;7(7):e41308. doi: 10.1371/journal.pone.0041308 (PMC3398900; doi:10.1371/journal.pone.0041308)
Supplement: Text S1 — Specific composition of each resource use category and assumptions made for their count. (PDF) [file pone.0041308.s003.pdf]

Supporting Information S1 – Text S1

***Title of the article:* Resource use and costs associated with coeliac disease before and after diagnosis in 3,646 cases: results of a UK primary care database analysis**

Text S1 – Specific composition of each resource use category and assumptions made for their count

The category 'consultations' included GP consultations in surgery, by telephone and at home/out-of-hours, and nurse consultations in surgery and at home/out-of-hours. The category 'nurse' included the following roles: community nurse, community psychiatric nurse, dietician, health visitor, midwife, other health care professional, physiotherapist, practice nurse, chiropodist and counsellor. Following a previous study [28], we counted more than one consulting record per day as one consultation only; in this way consultations were defined as contact days.

For the category of 'tests', with the advice of a GP we identified the medical codes for three main batches of tests referring to the electrolytes, blood count and liver function tests, respectively (codes available from the authors upon request). For each patient, each batch of tests (or any subgroup within the same batch) recorded on the same day was counted as one test only. The remaining types of tests were aggregated in the broad category 'Test-other'. More than one test record belonging to the latter category was considered as one test only, if it occurred on the same day. We excluded from our count tests that are routinely performed by a GP during a standard consultation (i.e. measuring weight, blood pressure and the like), which we assumed to be already included in the consultation cost, and if not excluded would have generated problems of double counting.

Following a previous study [27] the referrals included in the study were all to consultant outpatient clinics. Emergency admissions and attendance at Accident & Emergency were excluded. Normally GPs only record the first referral of a patient to a clinic for an episode of care and not follow-up appointments arranged by the clinic or even further appointments arranged by the GP during the same care event. Referrals were classified by their clinical specialty as recorded by the GP using the VAMP medical software (see Box 1 below). Similarly to Hodgson and Ellis [27], referrals to the specialties of Obstetrics, Genito-urinary, X-ray, Pathology and Others were excluded from the analysis, due to large variations over time in the way in which different GPs can record referrals to the above specialties within the VAMP Medical software. For example, it is possible to record X-ray and Pathology in a variety of ways using the VAMP medical software and most of them would be missed by an extract of referrals. Obstetrics care, to give another example, is frequently managed in cooperation with local midwives. As a consequence the referral procedure and the associated records vary from area to area [27]. For cost purposes a distinction was made between the NHS category 'Gastroenterology' on one side, and all the other categories included in the analysis on the other.

The number of prescriptions for each patient was calculated both in aggregate and stratified by 16 categories, namely each of the 15 Chapters of the BNF [21] and a further 'Miscellaneous' category, in which we included all those products in the BNF that are not classified in a BNF Chapter per se, e.g. some food supplements, dressings and appliances.

Box 1

**GPRD VAMP Medical Specialties**
General Surgical
General Medical
Orthopaedic
Rheumatology
Ear, Nose and Throat
Gynaecology
Obstetrics
Paediatric
Ophthalmology
Psychiatry
Geriatrics
Dermatology
Neurology
Genito-urinary
X-ray
Pathology
Others

Source: Hodgson and Ellis [27]
